# High-resolution Raman spectroscopy reveals compositional differences between pigmented incisor enamel and unpigmented molar enamel in *Rattus norvegicus*

**DOI:** 10.1038/s41598-023-38792-5

**Published:** 2023-07-29

**Authors:** Furqan A. Shah

**Affiliations:** Department of Biomaterials, Sahlgrenska Academy, University of Gothenburg, Gothenburg, Sweden

**Keywords:** Structural biology, Medical research, Dental diseases, Raman spectroscopy, Scanning electron microscopy, Enamel, Bone

## Abstract

Dental enamel is a peculiar biological tissue devoid of any self-renewal capacity as opposed to bone. Thus, a thorough understanding of enamel composition is essential to develop novel strategies for dental enamel repair. While the mineral found in bone and dental enamel is generally viewed as the biologically-produced equivalent of hydroxy(l)apatite, the formation of these bioapatites is controlled by different organic matrix frameworks—mainly type-I collagen in bone and amelogenin in enamel. In lower vertebrates, such as rodents, two distinct types of enamel are produced. Iron-containing pigmented enamel protects the continuously growing incisor teeth while magnesium-rich unpigmented enamel covers the molar teeth. Using high-resolution Raman spectroscopy, scanning electron microscopy, and energy dispersive X-ray spectroscopy, this work explores the differences in acid phosphate (HPO_4_^2−^), carbonate (CO_3_^2−^), hydroxyl (OH^−^), iron, and magnesium content of pigmented incisor enamel and unpigmented molar enamel of Sprague Dawley rats. Bundles of hydroxy(l)apatite nanowires comprise the enamel prisms, where prisms in pigmented enamel are wider and longer than those in unpigmented molars. In contrast to magnesium-rich unpigmented enamel, higher mineral crystallinity, and higher HPO_4_^2−^ and OH^−^ levels are hallmark features of iron-rich pigmented enamel. Furthermore, the apparent absence of iron oxides or oxy(hydroxides) indicates that iron is introduced into the apatite lattice at the expense of calcium, albeit in amounts that do not alter the Raman signatures of the PO_4_^3−^ internal modes. Compositional idiosyncrasies of iron-rich pigmented and nominally iron-free unpigmented enamel offer new insights into enamel biomineralisation supporting the notion that, in rodents, ameloblast function differs significantly between the incisors and the molars.

## Introduction

Dental enamel is arguably the toughest and most resilient biological tissue^1^. Despite the extraordinary mechanical properties that enable withstanding of fatigue and wear, dental enamel has limited ability for self-repair or renewal, unlike bone^2^. Though considered biologically produced analogues of hydroxy(l)apatite [Ca_5_(PO_4_)_3_OH], the apatites of bone and dental enamel are remarkably dissimilar^3^. In enamel, through pH-dependent supramolecular self-assembly, a protein called amelogenin plays a key role in guiding the elongated and highly oriented growth of apatite in enamel^4,5^. As a direct consequence of chemical gradients, the distinct core–shell structure and the residual stresses thus arising significantly impact the dissolution behaviour of human enamel crystallites^6^. In rodents, the continuously growing incisor teeth^7^ are protected by Fe-containing pigmented enamel while molar teeth are covered with Mg-rich unpigmented enamel^8^.

Raman spectroscopy can distinguish between different mineralised biological tissues, including enamel, dentine, cementum, and bone, and reveal vital information about the biological processes underpinning their formation and assembly^9^. Certain aspects of mineral composition, e.g., carbonate ion (CO_3_^2−^) incorporation, provide insights into the pathways of bioapatite formation^10^. The degree of carbonation (i.e., CO_3_^2−^ content), ultimately, affects the long-range order or mineral crystallinity^11^. Additionally, factors such as local pH influence the availability and incorporation of acid phosphate (HPO_4_^2−^) into the apatite lattice—i.e., with greater HPO_4_^2−^ being introduced under acidic conditions^12^. HPO_4_^2−^ containing phases such as octacalcium phosphate are frequently encountered in the mineralised dental biofilm^13^. And although human and bovine enamel are believed to contain about 5 wt% HPO_4_^2−^^14^, Raman studies of human premolar teeth have not been able to detect non-apatitic environments^15,16^.

Chemical and structural characterisation of biological tissues using Raman spectroscopy is often plagued by the intrinsic autofluorescence^17^. This process originates from various organic moieties^18^, but can be suppressed by methods such as deproteinisation with sodium hypochlorite (NaOCl)^19^. This work uses high-resolution Raman spectroscopy together with scanning electron microscopy (SEM) and energy dispersive X-ray spectroscopy (EDX) to probe the major compositional differences (particularly the HPO_4_^2−^, CO_3_^2−^, and OH^−^ environments) between two distinct types of enamel—pigmented incisor enamel (PIE) and unpigmented molar enamel (UME) in the rat (*Rattus norvegicus*), with and without deproteinisation. Furthermore, the Raman spectra are compared with geologic hydroxy(l)apatites from the RRUFF™ database^20^, such as the well-characterised and highly crystalline Holly Springs hydroxy(l)apatite^21,22^, which are typical reference materials in crystallographic studies of biogenic calcium phosphates^23^.

## Results

**Figure 1:**
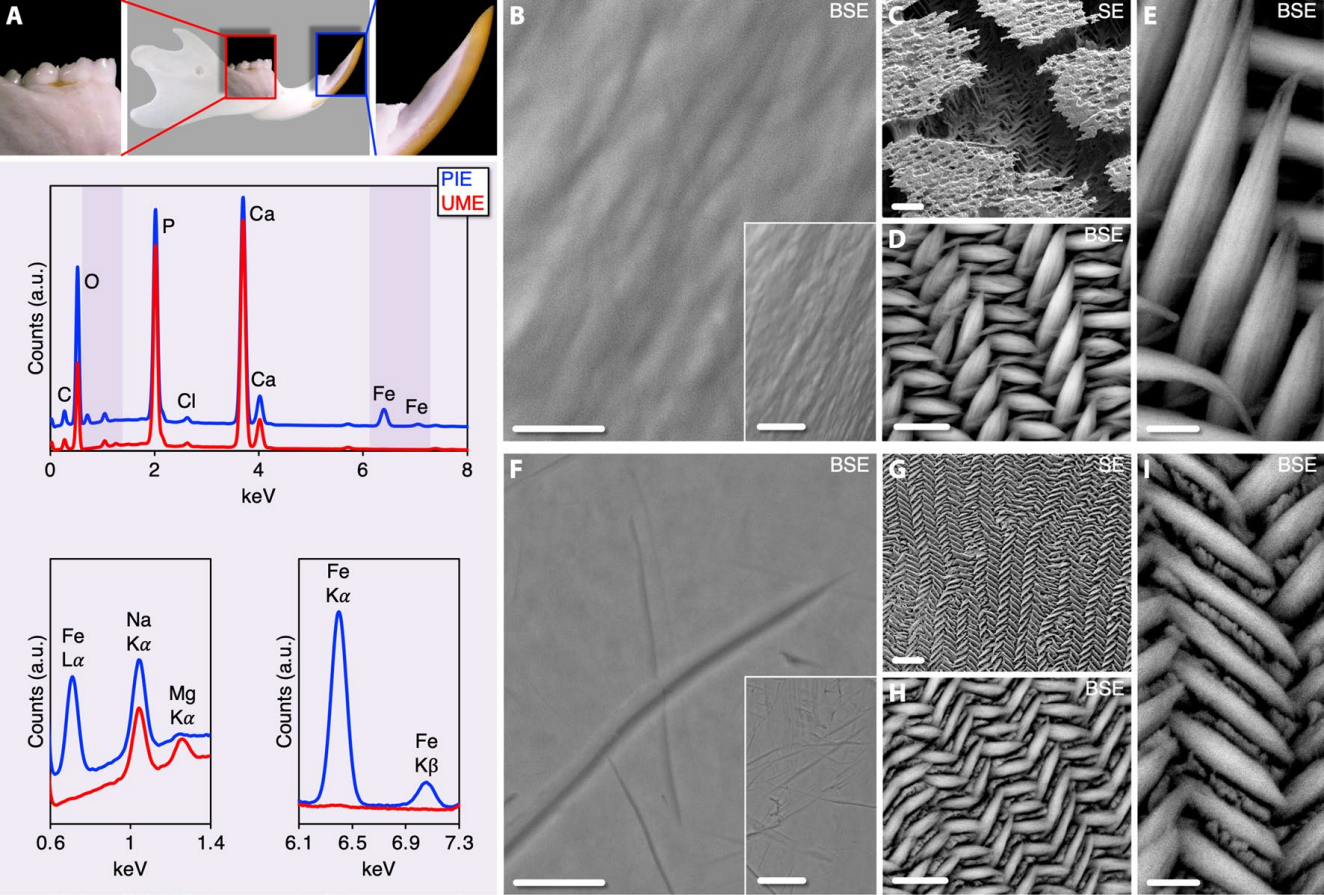
Elemental composition and microstructure. (**A**) PIE is Fe-rich while UME is Mg-rich (EDX). Spectra are normalised to the Ca Kα peak (at ~ 3.692 keV). (**B**–**I**) PIE surface is grainy (**B**) and UME surface is smooth (**F**). Insets: Respective lower magnifications (Scale bars = 25 µm). Enamel prisms in PIE (**C**–**E**) and UME (**G**–**I**) after H_3_PO_4_ etching. BSE = Backscattered electron mode. SE = Secondary electron mode. Scale bars in (**B**), (**C**), (**F**), and (**G**) = 10 µm, (**D**) and (**H**) = 5 µm, (**E**) and (**I**) = 2 µm.

The major X-ray emission lines Ca Kα (3.692 keV), Ca Kβ (4.013 keV), P Kα (2.014 keV), Fe Kα (6.403 keV), Fe Kβ (7.058 keV), Fe Lα (0.705 keV), and Mg Kα (1.254 keV) confirmed the differences in Ca/P ratio, Fe content, and Mg content between PIE and UME. The Ca/P ratio of UME (1.30 ± 0.02 at.%) is higher (*p* < 0.05) than PIE (1.19 ± 0.01 at.%). Similarly, the Mg/Ca ratio of UME (0.011 ± 0.001 at.%) is higher (*p* < 0.05) than PIE (0.002 ± 0.001 at.%). Whereas the Fe content (Fe/Ca ratio) of UME is negligible, PIE shows significant Fe enrichment (~ 0.15 ± 0.01 at.%), which results in higher (Ca + Mg + Fe)/P ratio (~ 4.2 ± 0.9 at.%), indicating the incorporation of iron at the expense of calcium (Fig. 1). Small amounts of Cl are also detected, which may occur by partial substitution of OH^−^^24^. Scanning electron microscopy (SEM) of the enamel surface reveals that UME is smooth while PIE is grainy in comparison. UME etches homogeneously with orthophosphoric acid (H_3_PO_4_) but PIE is mildly resistant to acid attack, as evident from the islands of an incompletely removed surface layer. When visualised after acid etching, bundles of hydroxy(l)apatite nanowires comprise the enamel prisms, where PIE prisms are wider and longer than UME prisms.

**Figure 2:**
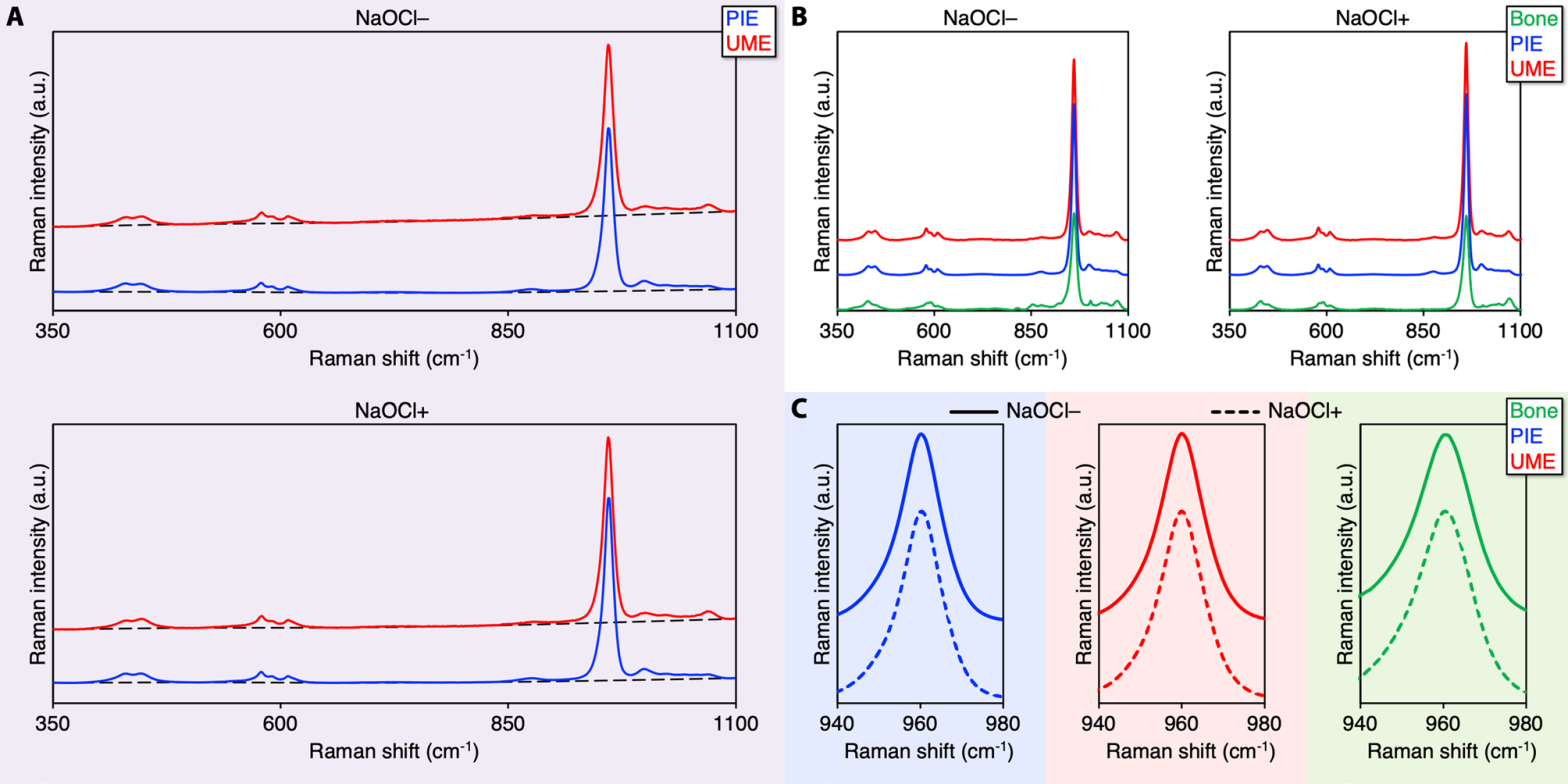
Background fluorescence and $\upnu$_1_ PO_4_^3−^ intensity. (**A**) UME generates stronger fluorescence than PIE, both before (NaOCl−) and after (NaOCl+) deproteinisation. Unprocessed spectra without baseline subtraction and cosmic ray removal (averaged Raman spectra). Broken lines indicate the background fluorescence profile. (**B**) Baseline corrected and normalised spectra. (**C**) $\upnu$_1_ PO_4_^3−^ band.

High-resolution Raman spectra were acquired over the 350–1100 cm^−1^ wavenumber range using the 2400 g mm^−1^ grating. In enamel and mandibular bone, the major Raman domains are $\nu$_2_ PO_4_^3−^ (symmetric bend) at 370–490 cm^−1^, $\nu$_4_ PO_4_^3−^ (asymmetric bend) at 545–635 cm^−1^, $\nu$_1_ PO_4_^3−^ (symmetric stretch) at 960 cm^−1^, and $\nu$_3_ PO_4_^3−^ (asymmetric stretch) at 1015–1095 cm^−1^^25^. Estimated from the difference in the background signal over the 350–1100 cm^−1^ spectral range, UME generates 381 ± 18% higher fluorescence than PIE, which decreases to 262 ± 9% after deproteinisation **(**Fig. 2**)**. Normalised to the polarisation-insensitive $\nu$_2_ PO_4_^3−^ band ^26^, the $\nu$_1_ PO_4_^3−^ band (~ 960 cm^−1^) of enamel is substantially stronger than bone.

**Figure 3:**
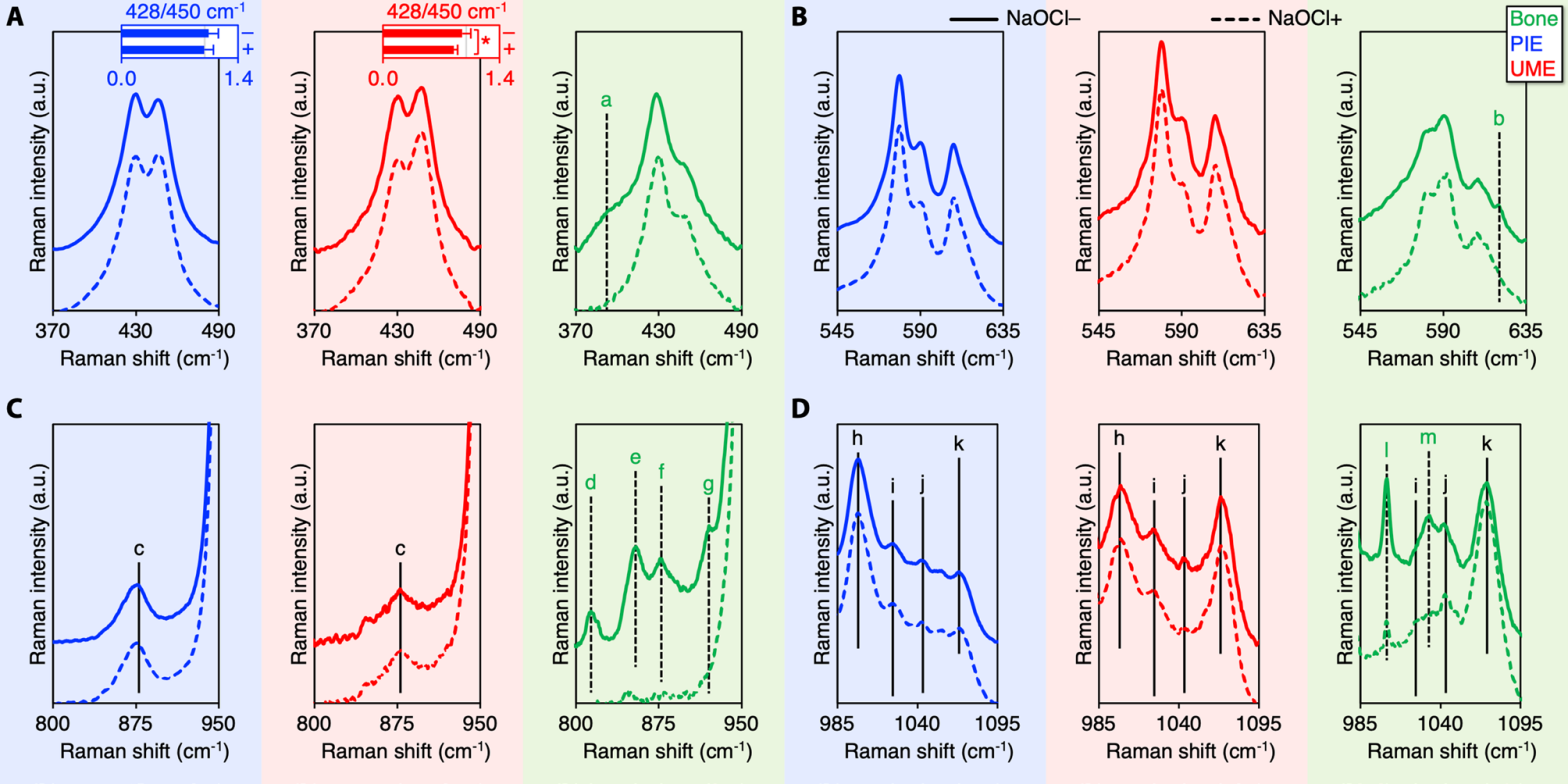
HPO_4_^2−^, CO_3_^2−^, and the organic matrix in PIE, UME, and bone. (**A**) $\upnu$_2_ PO_4_^3−^ band. Insets: 428/450 cm^−1^ ratios before (NaOCl−) and after (NaOCl+) deproteinisation. (B) $\upnu$_4_ PO_4_^3−^ band. (**C**) HPO_4_^2−^, proline (at 853 cm^−1^), and hydroxyproline (at 876 cm^−1^) bands. (**D**) $\upnu$_3_ PO_4_^3−^, $\upnu$_1_ HPO_4_^2−^, $\upnu$_1_ CO_3_^2−^, and phenylalanine (at 1004 cm^−1^) bands. Features labelled a, b, d, e, f, g, l, and m represent the organic matrix of bone, c and h indicate HPO_4_^2−^, i and j are assigned as $\upnu$_3_ PO_4_^3−^, and k is $\upnu$_1_ CO_3_^2−^.

The $\nu$_2_ PO_4_^3−^ band consists of sub-components at 428 cm^−1^ and 450 cm^−1^, of which the 428 cm^−1^ sub-component tends to be stronger in carious enamel^15^ and synthetic hydroxy(l)apatite^27^ (Fig. 3). The 428/450 cm^−1^ ratios of PIE (1.05 ± 0.12) and UME (0.95 ± 0.10) are close to unity and comparable (*p* > 0.15). Upon deproteinisation, the 428/450 cm^−1^ ratio of UME (0.85 ± 0.05) shows a minor decrease (*p* = 0.026) and is also lower (*p* = 0.013) than that of PIE after deproteinisation (1.00 ± 0.11). Bone also shows decreased 428/450 cm^−1^ ratio upon deproteinisation, from ~ 1.63 to ~ 1.58, and the organic matrix is lost. PIE and UME display comparable $\nu$_4_ PO_4_^3−^ band profiles. Among the $\nu$_4_ PO_4_^3−^ band sub-components (580 cm^−1^, 590 cm^−1^, and 607 cm^−1^), the 580 cm^−1^ sub-component in enamel is the strongest while it is significantly weaker for bone and often less intense than the 590 cm^−1^ sub-component. Furthermore, a shoulder is observed at 621 cm^−1^ in bone only prior to deproteinisation, and is therefore assigned to the organic matrix^25^.

**Figure 4:**
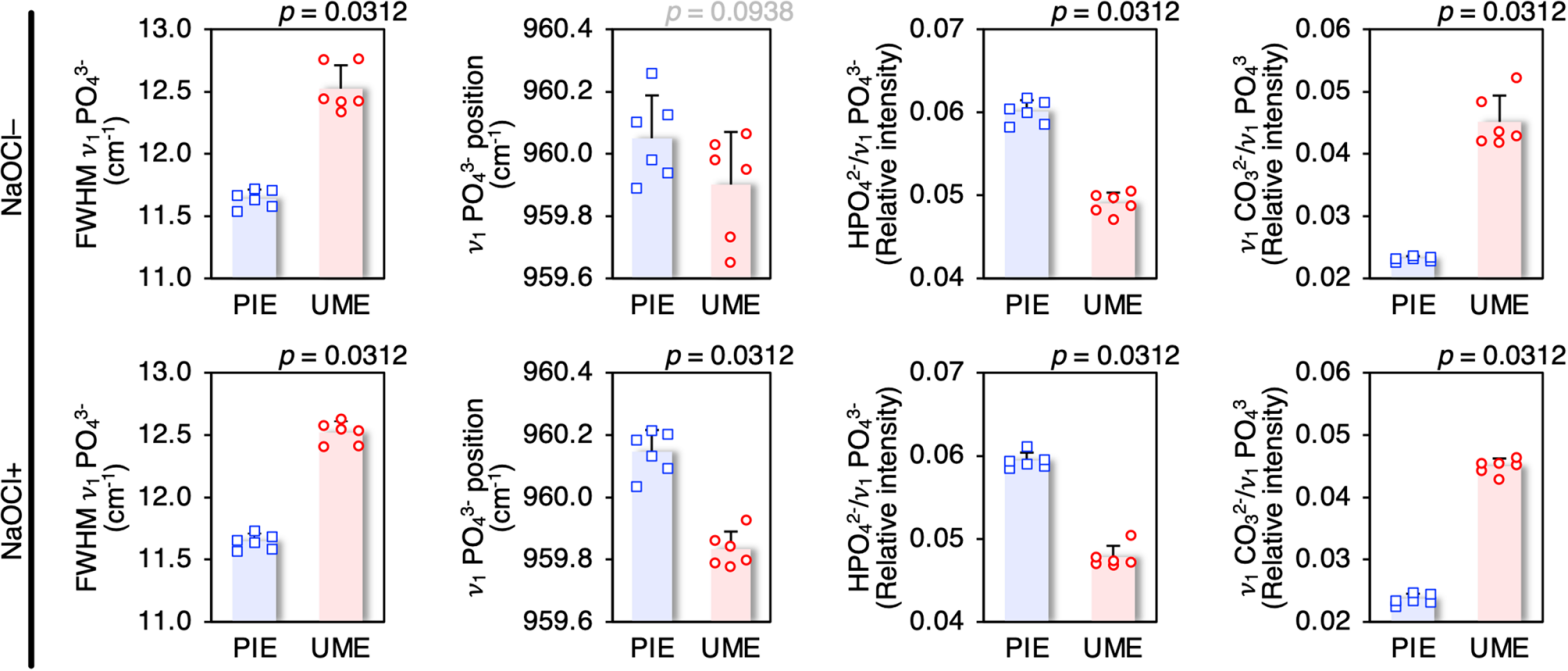
Compositional differences between PIE and UME. Mineral crystallinity, $\upnu$_1_ PO_4_^3−^ peak position, HPO_4_^2−^ content (1000/960 cm^−1^), and CO_3_^2−^ content (1070/960 cm^−1^) of PIE and UME.

Mineral crystallinity, i.e., the inverse full-width at half-maximum (FWHM) of the $\nu$_1_ PO_4_^3−^ band, of enamel is higher than bone **(**Fig. 4**)**. Upon deproteinisation, the FWHM $\nu$_1_ PO_4_^3−^ of PIE (~ 11.65 cm^−1^) and UME (~ 12.5 cm^−1^) remains unchanged while that of bone decreases by ~ 3.4% from 15.97 to 15.43 cm^−1^. Compared to PIE, the $\nu$_1_ PO_4_^3−^ band of UME is shifted to lower wavenumbers. This shift in $\nu$_1_ PO_4_^3−^ position (~ 0.31 ± 0.06 cm^−1^) becomes particularly evident after deproteinisation (*p* = 0.0312). Broad features at ~ 878 cm^−1^ and 1000 cm^−1^ are attributable to HPO_4_^2−^ groups in enamel^28^. The HPO_4_^2−^ content (1000/960 cm^−1^ ratio) of UME is lower (*p* = 0.0312) than PIE. The $\nu$_3_ PO_4_^3−^ sub-components at ~ 1027 cm^−1^ and ~ 1046 cm^−1^ are observed consistently for enamel and bone. The $\nu$_1_ CO_3_^2−^ band is centred at ~ 1069 cm^−1^ for enamel and ~ 1071 cm^-1^ for bone. The CO_3_^2−^ content ($\nu$_1_ CO_3_^2−^/$\nu$_1_ PO_4_^3−^ intensity ratio) of bone is substantially higher than enamel, increasing from ~ 0.09 to ~ 0.12 upon deproteinisation. Furthermore, the CO_3_^2−^ content of UME is higher than PIE (*p* = 0.0312).

**Figure 5:**
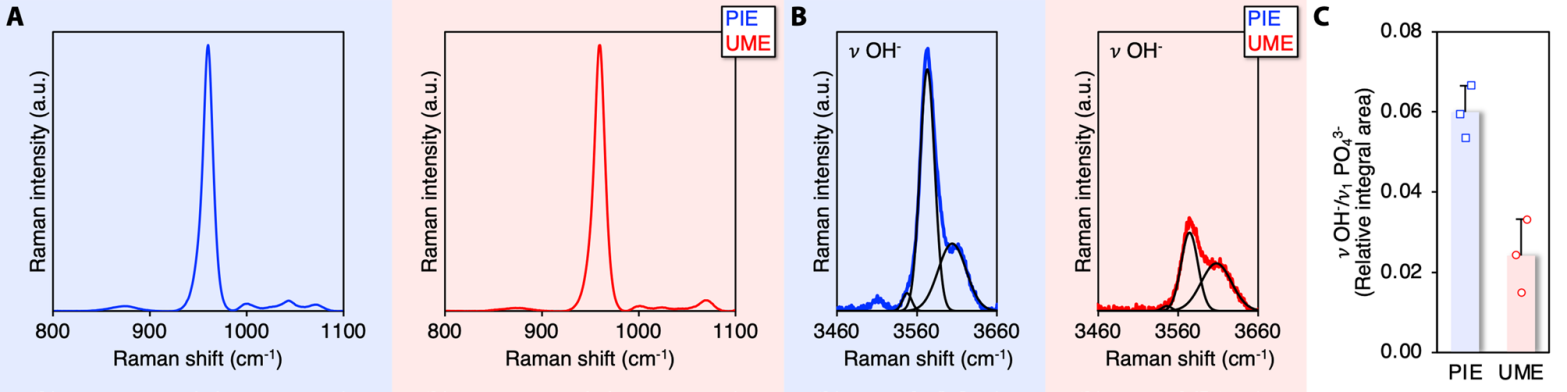
Hydroxl (OH^−^) content in PIE and UME (1800 g mm^−1^ grating). (**A**) Baseline corrected and normalised spectra. (**B**) $\upnu$ OH^−^ at 3573 cm^−1^ and Ca(OH)_2_ at 3618 cm^−1^. (**C**) $\upnu$ OH^−^ content (3573/960 cm^−1^).

Extended-range Raman spectra were acquired over the 800–3700 cm^−1^ wavenumber range using the 1800 g mm^−1^ grating (Fig. 5). The $\nu$ OH^−^ region (3460–3660 cm^−1^) shows an asymmetrical band. The OH^−^ content, taken as the integral area ratio between $\nu$ OH^−^ (~ 3573 cm^−1^) and $\nu$_1_ PO_4_^3−^ (930–990 cm^−1^), of PIE is ~ 148% greater than UME. The feature at ~ 3618 cm^−1^ is assigned as Ca(OH)_2_.

**Figure 6:**
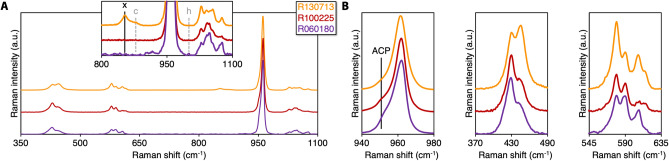
Geologic hydroxy(l)apatites. (**A**) Overview and detail of the 800–1100 cm^−1^ region of R130713 (Wessels mine, Northern Cape Province, South Africa); R100225 (Sapo mine, Minas Gerais, Brazil); and R060180 (Holly Springs, Georgia, USA). Inset: An unidentified peak (feature labelled x) is noted at ~ 853 cm^−1^ for RRUFF R130713. HPO_4_^2−^ (features labelled c and h) is absent for all geologic hydroxy(l)apatites. (**B**) $\upnu$_1_ PO_4_^3−^, $\upnu$_2_ PO_4_^3−^, and $\upnu$_4_ PO_4_^3−^ bands.

Raman spectra of enamel and bone were compared to three geologic hydroxy(l)apatites. Remarkable similarities are noted between enamel (PIE and UME) and hydroxy(l)apatite from the Wessels mine (Northern Cape Province, South Africa. RRUFF: R130713) (Fig. 6). An unidentified feature is seen at ~ 853 cm^−1^ for R130713, while the characteristic peaks for HPO_4_^2−^ at 878 cm^−1^ and 1000 cm^−1^ are absent. On the other hand, hydroxy(l)apatite from the Sapo mine (Minas Gerais, Brazil. RRUFF: R100225) and Holly Springs (Georgia, USA. RRUFF: R060180) more closely resemble bone. Small amounts of amorphous calcium phosphate, indicated by a shoulder at ~ 950 cm^−1^^29^, are present in all of the geologic hydroxy(l)apatites.

## Discussion

Better understanding of enamel composition is essential to develop biomimetic and bioinspired strategies for enamel repair^30^. Some of the recent and highly divergent approaches to repair enamel include protein order/disorder-guided hierarchical mineralised structures^31^ and epitaxially-grown hydroxy(l)apatite crystals^32^. In mineralised biological systems, the presence of iron is often associated with high strength—a prime example being the teeth of the common limpet, thought to be the strongest known biomaterial, where iron-containing filamentous crystals of Goethetite [α-FeO(OH)] comprise the reinforcing phase^33^. Likewise, the presence iron contributes to the overall mechanical properties of rodent pigmented enamel^8^. In the northern short-tailed shrew (*Blarina brevicauda*), iron pigmentation is not confined to the incisors but exists as a general feature of high stress areas on most teeth^34^. Curiously, iron pigmentation of the dental enamel has also been observed in mammalian species as early as the late Cretaceous period^35^.

In rodents, differences in enamel architecture between unpigmented molar enamel, which forms during embryogenesis, and pigmented incisor enamel, which forms during post-natal life, relate to genetic control of ameloblast differentiation involving distinct mechanisms at these distinct phases of life^36^. Autophagy related 7 (ATG7) protein is essential for the secretion of iron from ameloblasts^37^. Moreover, iron deficiency leads to gross loss of pigmentation and enamel hypoplasia/aplasia^38^. Although amelogenin plays a fundamental role in achieving the precise crystal habit, the enzyme matrix metalloproteinase-20 prevents protein occlusion inside apatite crystals^39^.

Fe enrichment of pigmented enamel is possible through partial substitution of Ca^2+^ without major changes in PO_4_^3−^ internal modes^40^, although a constriction in lattice parameters is expected^41^. PIE appears to resist acid attack, which has earlier been attributed to the presence of Ca^2+^ and Mg^2+^ substituted ferrihydrite^8^. However, in the present work, micro-Raman spectroscopy has not revealed evidence of iron oxides or oxy(hydroxides) in PIE^42^. And though it not straightforward to ascertain the oxidation state of Fe (Fe^2+^ or Fe^3+^) from EDX, alone, Fe-L_2,3_ electron energy-loss near-edge structure (ELNES) of pigmented Fe-rich enamel from the rodent *Myocaster coypus* suggests a predominantly Fe^3+^ state^43^. Under the assumption that Fe occupies Ca sites in iron-pigmented enamel, the Fe/Ca ratio of 0.15 equates to ~ 13% Ca substitution and therefore ~ 5.15% mass difference. Ab initio calculations of ^42^Ca isotopic substitution for ^40^Ca, which equates to ~ 5% mass difference at the Ca sites, have revealed that the expected Raman shifts for vibrational modes above ~ 600 cm^−1^ (for example the $\nu$_1_ PO_4_^3−^ band) do not exceed ~ 1 cm^−1^^44^. Here, high-resolution Raman spectroscopy reveals this very small shift in $\nu$_1_ PO_4_^3−^ peak position for the first time. In unpigmented enamel, Mg^2+^ accumulates within intergranular regions of amorphous calcium phosphate^6,45^. Compared to rat molars, as reported here, the Mg content at the surface of human molars is nearly twice as much at the enamel surface and progressively increases towards the dentinoenamel junction^46^.

Fourier transform infrared spectroscopy studies have suggested the presence of non-apatitic environments (e.g., HPO_4_^2−^ groups) in porcine enamel^47^. Here, high-resolution Raman spectroscopy confirms the presence of HPO_4_^2−^ in both pigmented and unpigmented rat enamel. HPO_4_^2−^ is thought to be a precursor phosphate source for enamel apatite^48^. Therefore, detection of higher HPO_4_^2−^ at the surface of PIE (vs. UME) may be a function of tissue age, as has been reported across different developmental stages of porcine enamel^49^. It has been suggested that acidic conditions favour the fast growth of highly crystalline hydroxy(l)apatite by dissociating calcium phosphate aggregates into Ca^2+^ and PO_4_^3−^ ions, which would otherwise block crystal growth and lead to lower crystallinity^50^. If the higher crystallinity and greater HPO_4_^2−^ content of PIE (vs. UME) can be explained by a more acidic environment, it must be determined how this acidic pH is regulated, e.g., if it is biologically driven. Removal of OH^−^ from the local environment through incorporation into the apatite lattice, also more abundant in PIE than in UME, further points towards the presence of acidic conditions. Nevertheless, the OH^−^ content of PIE is lower than values of human and boar enamel reported by Pasteris and co-workers^27^. The anticorrelation between CO_3_^2−^ content and crystallinity with little apparent influence of HPO_4_^2−^ warrants further investigation and raises the question whether crystallinity correlates with CO_3_^2−^ only.

Organic contamination of UME to a greater extent than PIE is hardly surprising since the latter is continually lost to wear and replaced by pristine mineral. Change in the 428/450 cm^−1^ ratio of UME, from ~ 1 (indicating high symmetry of PO_4_^3−^ groups) to 0.85 after deproteinisation, suggests a reduction in symmetry and that UME is more susceptible than PIE to alterations. The detection of Ca(OH)_2_ points towards the presence of CaO, which readily reacts with atmospheric humidity^51^. Finally, simultaneous increases in mineral crystallinity and CO_3_^2−^ content of bone upon deproteinisation are artefactual and imply loss of recently deposited extracellular matrix and poorly crystalline mineral at the bone surface^52^.

In summary, the chemical contrasts between pigmented and unpigmented enamel in rodents, including HPO_4_^2−^ content, CO_3_^2−^ content, mineral crystallinity, reflect ameloblast function and point towards putative differences in the specific local environmental conditions (e.g., the interplay between pH and the HCO_3_^-^ buffer system^53^) of the organic extracellular matrix and matrix metalloproteinase-20 activity during enamel biomineralisation. While the precise functional role of iron in tooth development remains unclear, iron accumulation in rodent incisors (and the presence of iron in mature ameloblasts) is related to the continuously erupting nature of this tooth^54^. This characteristic feature of rodent incisors also serves to explain the higher HPO_4_^2−^ content of PIE (vs. UME). On the other hand, the high CO_3_^2−^ content of UME is attributed to B-type substitution (i.e., CO_3_^2−^ for PO_4_^3−^) typical of biological apatites^55^, and contributes to lower crystallinity together with Mg^2+^^56^.

## Materials and methods

**Figure 7:**
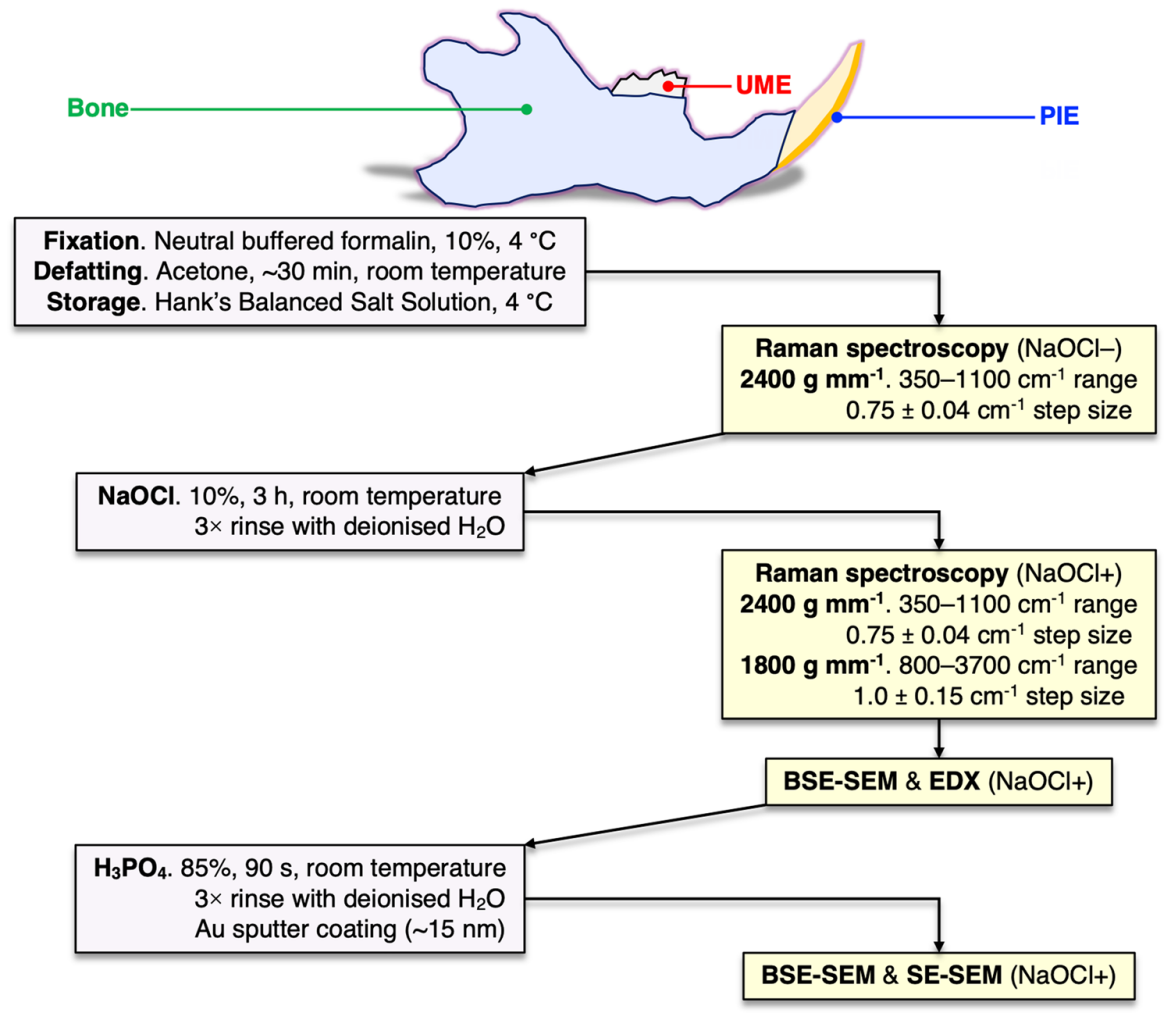
Experimental workflow.

Hemi-mandibles of adult Sprague Dawley rats, obtained as part of an unrelated study, were fixed in 10% neutral buffered formalin, defatted in acetone (~ 30 min), and stored in Hank’s Balanced Salt Solution (Gibco™) at 4 °C (Fig. 7). The organic constituents were removed by exposure to 10% NaOCl (3 h at room temperature). The experiment was approved by the local Animal Ethics Committee at the University of Gothenburg (Dnr 5.8.18-12983/2021) and performed in accordance with relevant guidelines and regulations.

### Scanning electron microscopy

Backscattered electron (BSE) imaging and energy dispersive X-ray spectroscopy (EDX) were performed in a Quanta 200 environmental SEM (FEI Company, The Netherlands) equipped with an INCA EDX system (Oxford Instruments GmbH, Wiesbaden, Germany) operated at 1 Torr water vapour pressure, 20 kV accelerating voltage, 0–10 keV spectral energy range, and 10 mm working distance (NaOCl+ ; 1 spot per sample, *n* = 6). To visualise enamel prisms, deproteinised hemi-mandibles were etched with 85% H_3_PO_4_ (90 s at room temperature) and Au sputter coated (~ 15 nm thickness). Secondary electron (SE) imaging was performed in an Ultra 55 FEG SEM (Leo Electron Microscopy Ltd, UK) operated at 5 kV accelerating voltage.

### Micro-Raman spectroscopy

Micro-Raman spectroscopy was performed using a confocal Raman microscope (Renishaw inVia Qontor) equipped with a 633 nm laser and LiveTrack™ focus-tracking technology^57^. The laser was focussed down on to PIE (labial aspect), UME (buccal aspect), and bone (mandibular ramus) surface using a × 50 objective. The Raman scattered light was collected using a Peltier cooled CCD deep depletion NIR enhanced detector. Using the 2400 g mm^−1^ grating (348–1104 cm^−1^ wavenumber range, step size of 0.75 ± 0.04 cm^−1^), Raman spectra were obtained from enamel (9 spots per sample) at 8 s (NaOCl−) or 4 s (NaOCl+) integration time and 10 accumulations, and from bone (one spot per sample) at 10 s (NaOCl−) or 5 s (NaOCl+) integration time and 20 accumulations. Using the 1800 g mm^−1^ grating (800–3700 cm^−1^ wavenumber range, SynchroScan wide-range scanning mode; step size of 1.0 ± 0.15 cm^−1^), Raman spectra were obtained from enamel (3 spots per sample, NaOCl+) at ~ 60 s integration time and 10 accumulations. The laser power at the sample was ~ 15 mW. Background subtraction and cosmic ray removal were performed using *intelligent spline* fitting in Renishaw WiRE 5.4 software.

### Statistical analysis

The Wilcoxon signed-rank test was used for statistical analysis. Mean values ± standard deviations are presented and *p* values < 0.05 were considered statistically significant.

## Data Availability

The datasets used and/or analysed during the current study available from the corresponding author on reasonable request.
